# Supplementary material for: AMPK-Nrf2 Signaling Pathway in Phrenic Motoneurons following Cervical Spinal Cord Injury
Source: Antioxidants (Basel). 2022 Aug 26;11(9):1665. doi: 10.3390/antiox11091665 (PMC9495920; doi:10.3390/antiox11091665)
Supplement: Supplementary file 1 [file antioxidants-11-01665-s001.zip › antioxidants-1862945-supplementary.pdf]

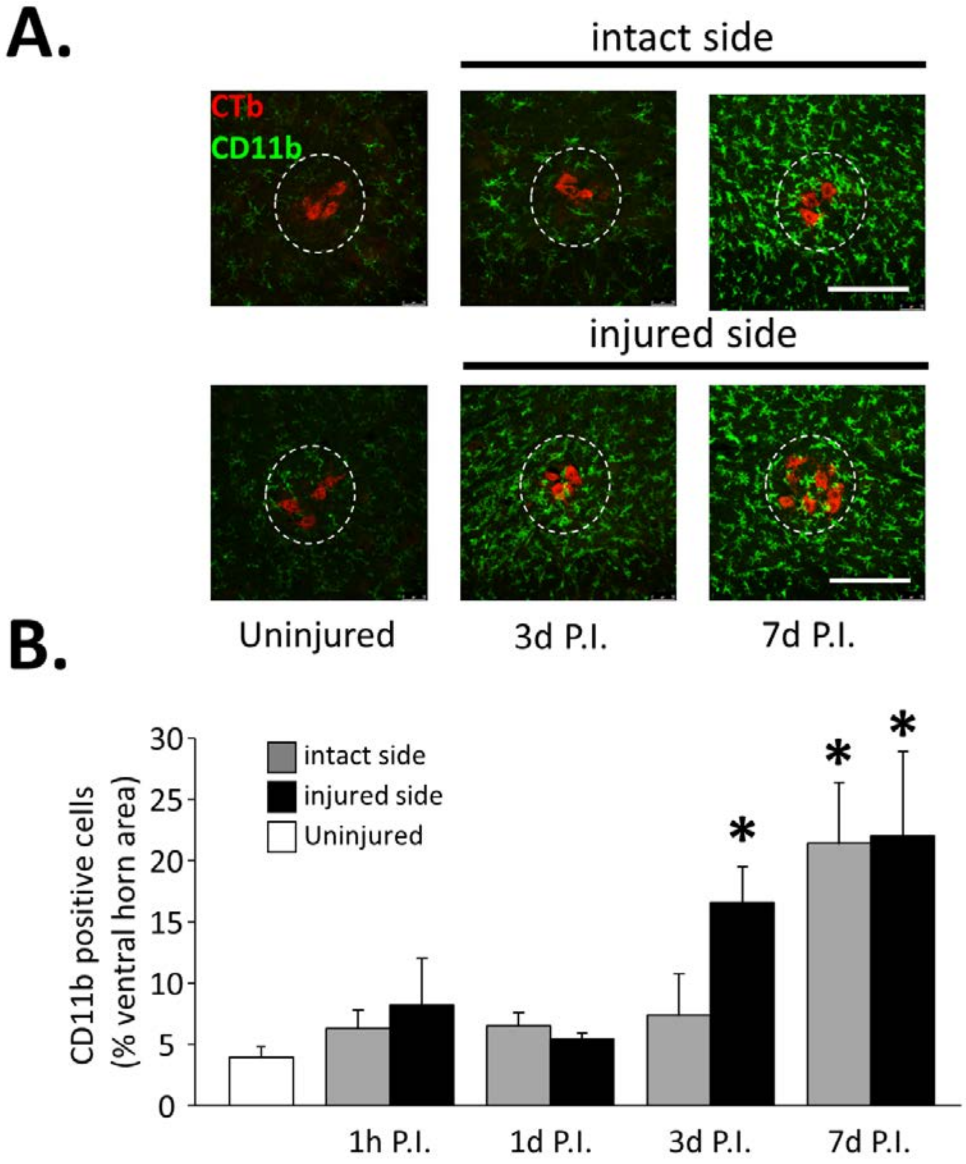

Figure S1: CD11b expression around phrenic motoneurons following C2 hemisection: (A) representative images showing CD11b positive cells around phrenic motoneurons labeled with CTB in uninjured animals and around denervated phrenic motoneurons in C2 hemisected rats following 3-day (d) and 7d post-injury (P.I.); (B) Percentage of CD11b positive cells in the ventral part of the C3-C6 spinal cord for uninjured animals, and intact and injured sides of C2 hemisected animals 1h, 1d, 3d and 7 following injury. * compared to uninjured group, $p < 0.05$.

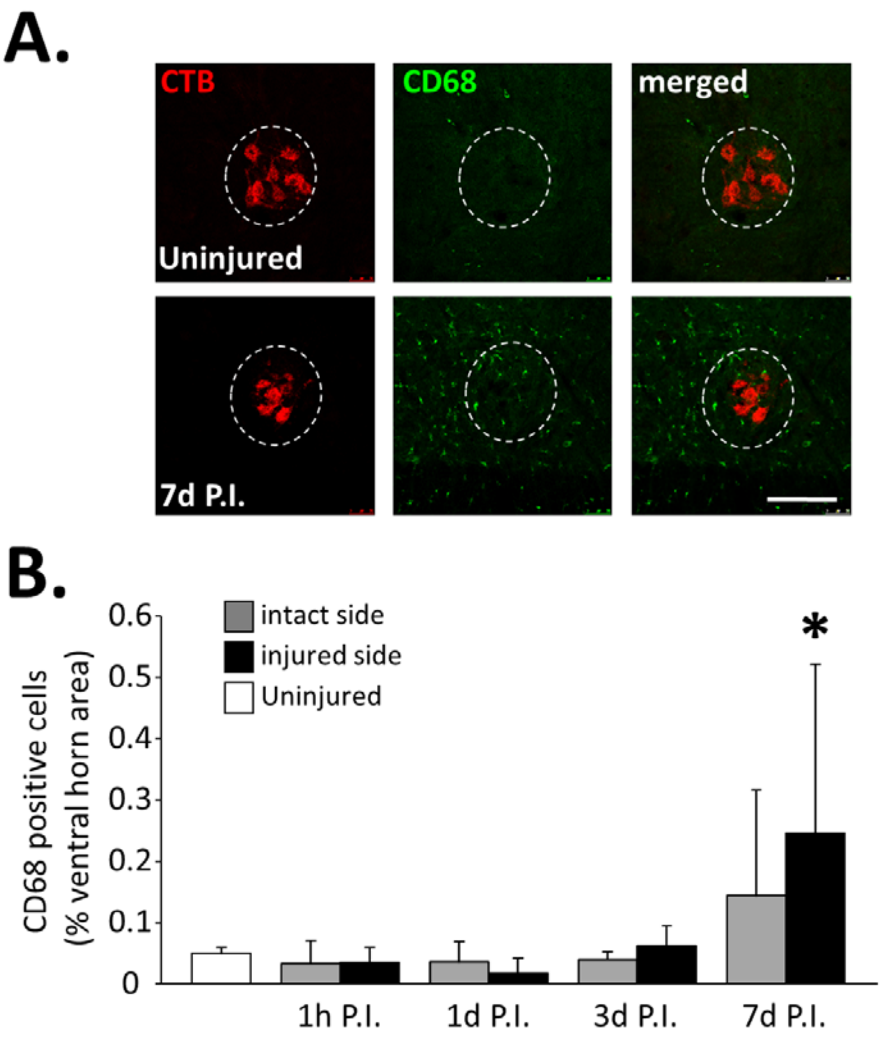

Figure S2: CD68 expression around phrenic motoneurons following C2 hemisection: (A) representative images showing CD68 positive cells around phrenic motoneurons labeled with CTB in uninjured animals and around denervated phrenic motoneurons in C2 hemisected rats following 7-day (d) post-injury (P.I.); (B) Percentage of CD68 positive cells in the ventral part of the C3-C6 spinal cord for uninjured animals, and intact and injured sides of C2 hemisected animals 1h, 1d, 3d and 7 following injury. * compared to 1d P.I. corresponding side, $p < 0.05$.
